# Epigenetic Repression of Chloride Channel Accessory 2 Transcription in Cardiac Fibroblast: Implication in Cardiac Fibrosis

**DOI:** 10.3389/fcell.2021.771466

**Published:** 2021-11-12

**Authors:** Tinghui Shao, Yujia Xue, Mingming Fang

**Affiliations:** ^1^Key Laboratory of Targeted Intervention of Cardiovascular Disease and Collaborative Innovation Center for Cardiovascular Translational Medicine, Department of Pathophysiology, Nanjing Medical University, Nanjing, China; ^2^Center for Experimental Medicine, Jiangsu Health Vocational College, Nanjing, China; ^3^Institute of Biomedical Research, Liaocheng University, Liaocheng, China

**Keywords:** transcriptional regulation, epigenetics, histone deacetylation, histone deacetylase, cardiac fibroblast, myocardial fibrosis

## Abstract

Cardiac fibrosis is a key pathophysiological process that contributes to heart failure. Cardiac resident fibroblasts, exposed to various stimuli, are able to *trans-*differentiate into myofibroblasts and mediate the pro-fibrogenic response in the heart. The present study aims to investigate the mechanism whereby transcription of chloride channel accessory 2 (Clca2) is regulated in cardiac fibroblast and its potential implication in fibroblast-myofibroblast transition (FMyT). We report that Clca2 expression was down-regulated in activated cardiac fibroblasts (myofibroblasts) compared to quiescent cardiac fibroblasts in two different animal models of cardiac fibrosis. Clca2 expression was also down-regulated by TGF-β, a potent inducer of FMyT. TGF-β repressed Clca2 expression at the transcriptional level likely *via* the E-box element between −516 and −224 of the Clca2 promoter. Further analysis revealed that Twist1 bound directly to the E-box element whereas Twist1 depletion abrogated TGF-β induced Clca2 *trans-*repression. Twist1-mediated Clca2 repression was accompanied by erasure of histone H3/H4 acetylation from the Clca2 promoter. Mechanistically Twist1 interacted with HDAC1 and recruited HDAC1 to the Clca2 promoter to repress Clca2 transcription. Finally, it was observed that Clca2 over-expression attenuated whereas Clca2 knockdown enhanced FMyT. In conclusion, our data demonstrate that a Twist1-HDAC1 complex represses Clca2 transcription in cardiac fibroblasts, which may contribute to FMyT and cardiac fibrosis.

## Introduction

Cardiac fibrosis is generally considered an adaptive response to adversarial stimuli when the heart is exposed to various injuries. A specialized cell type termed “myofibroblast,” typically absent from the normal myocardium under physiological conditions, emerges following injuries and mediates the fibrogenic response (Tomasek et al., 2002). On the one hand, myofibroblasts are capable of muscle-like contraction owing to the acquisition of the expression of genes encoding contractile proteins (e.g., α-SMA). Contraction by myofibroblasts facilitates wound healing and prevents the incidence of cardiac rupture (Talman and Ruskoaho, 2016). On the other hand, myofibroblasts produce multiple extracellular matrix proteins (e.g., type I collagen, type III collagen, fibronectin) to promote ventricular remodeling and maintain myocardial integrity (van den Borne et al., 2010). After the recession of injurious stimuli, myofibroblasts are no longer needed and thus must be removed or resolved to terminate cardiac fibrogenesis. On the contrary, persistent presence of myofibroblasts in the heart or failure of resolution often leads to aberrant and averse cardiac remodeling and increased rigidity of the myocardium dampening heart function. In fact, cardiac fibrosis is frequently observed and associated with poor diagnosis in patients with heart failure (Gonzalez et al., 2018).

The origin of myofibroblasts in the stressed heart was a subject matter of great controversy and remained elusive prior to the development and utilization of genetic lineage tracing technique. It has been proposed that microvascular endothelial cells (Zeisberg et al., 2007), epicardial epithelial cells (Zhou et al., 2010), myelocytic fibrocyte (Mollmann et al., 2006), and perivascular mesenchymal cells (Kramann et al., 2015) may *trans-*differentiate into myofibroblasts *in vitro* and/or *in vivo* under different conditions. Landmark studies from the Molkentin laboratory (Kanisicak et al., 2016) and the Evans laboratory (Moore-Morris et al., 2014), aided by lineage tracing, have unequivocally demonstrated that cardiac resident fibroblasts are the predominant source of mature myofibroblasts and become the effector cell type of cardiac fibrosis following cardiac injury *via* fibroblast-myofibroblast transition (FMyT). Further analysis has revealed that cardiac myofibroblasts can be labeled by periostin (encoded by *postn*), a matricellular protein that can function as a ligand for integrins to promote cell migration (Stempien-Otero et al., 2016). One of the most convincing pieces of evidence that supports the pivotal role of myofibroblasts in cardiac fibrosis is provided by Kaur et al. (2016) who demonstrate that elimination of periostin-positive cells (mature myofibroblasts), by diphtheria toxin mediated killing, abrogates aberrant fibrogenic response and preserves heart function after myocardial infarction. Despite these advances, many transcriptional events taking place during FMyT remain to be investigated in detail.

Chloride channel accessory 2 (Clca2) belongs to the family of calcium sensitive chloride conductance proteins or regulators (Jentsch and Pusch, 2018). Clca2 plays versatile pathophysiological roles by regulating multiple distinct yet interconnected cellular processes including proliferation (Walia et al., 2009), differentiation (Ramena et al., 2016), migration (Sasaki et al., 2012), and apoptosis (Seltmann et al., 2018). Early characterization of Clca2 protein structure and expression pattern indicated that Clca2 might be a regulator of cystic fibrosis (Gruber et al., 1999). More recently, Walia et al. (2012) have reported that Clca2 expression can be down-regulated by TGF-β, one of the most potent inducer of tissue fibrogenesis, in epithelial cells. In mammalian cells, gene expression is acutely influenced by the epigenetic machinery. Epigenetics mechanisms are heritable phenotypic changes that do not involve alterations in the DNA sequence; these mechanisms play an important role in a wide spectrum of human diseases (Surguchov et al., 2017). These observations prompted us to investigate whether and, if so, how Clca2 expression might be regulated in the process of cardiac FMyT. We report here that Clca2 is transcriptionally repressed by a Twist1-HDAC1 epigenetic complex in cardiac fibroblasts by pro-fibrogenic stimuli. In addition, Clca2 is able to modulate TGF-β induced FMyT *in vitro*.

## Materials and Methods

### Animals

All animal protocols were reviewed and approved the intramural Ethics Committee on Humane Treatment of Laboratory Animals of Jiangsu Health Vocational College. The mice were maintained in an SPF environment with 12 h light/dark cycles and libitum access to food and water. Cardiac fibrosis was induced by permanent ligation of left-anterior descending (LAD) coronary artery or transverse aortic constriction (TAC) as previously described (Yang et al., 2017; Liu et al., 2021b, c).

### Cell Culture, Plasmids, Transient Transfection, and Reporter Assay

Primary cardiac fibroblasts were isolated and maintained in DMEM supplemented with 10% FBS as previously described (Gao et al., 2020; Liu et al., 2020; He et al., 2021; Zhao et al., 2021). Mouse embryonic fibroblasts (MEFs) were isolated and maintained in DMEM supplemented with 10% FBS as previously described (Angrisani et al., 2021). Clca2 promoter-luciferase construct was made by amplifying genomic DNA spanning the proximal promoter and the first exon of Clca2 gene (−1100/ + 91) and ligating into a pGL3-basic vector (Promega). Truncation mutants were made using a QuikChange kit (Thermo Fisher Scientific, Waltham, MA, United States) and verified by direct sequencing. Small interfering RNAs were purchased from Dharmacon. Transient transfection was performed with Lipofectamine 2000. Cells were harvested 48 h after transfection and reporter activity was measured using a luciferase reporter assay system (Promega) as previously described (Kong et al., 2021a, c; Liu et al., 2021c; Zhang et al., 2021). MS-275 and MC-1568 were purchased from Selleck. Mouse recombinant TGF-β was purchased from R&D.

### Protein Extraction, Immunoprecipitation and Western Blot

Whole cell lysates were obtained by re-suspending cell pellets in RIPA buffer (50 mM Tris pH7.4, 150 mM NaCl, 1% Triton X-100) with freshly added protease inhibitor (Roche) as previously described (Chen et al., 2020a,b,c; Wu X. et al., 2020; Yang et al., 2020b; Zhang et al., 2020; Chen B. et al., 2021; Dong et al., 2021). Nuclear proteins were extracted using the NE-PER Kit (Pierce) following manufacturer’s recommendation. Specific antibodies or pre-immune IgGs were added to and incubated with cell lysates overnight before being absorbed by Protein A/G-plus Agarose beads (Santa Cruz). Precipitated immune complex was released by boiling with 1X SDS electrophoresis sample buffer. Western blot analyses were performed with anti-Clca2 (Proteintech, 19273-1, 1:500), anti-α-SMA (Sigma, A2547, 1:8000), anti-collagen type I (Proteintech, 14695-1, 1:2000), anti-Twist1 (Proteintech, 25465-1, 1:500), anti-HDAC1 (Santa Cruz, sc-7872, 1:1000), anti-HDAC2 (Santa Cruz, sc-7899, 1:1000), anti-HDAC3 (Santa Cruz, sc-11417, 1:1000), anti-HDAC8 (Santa Cruz, sc-11405, 1:1000), anti-FLAG (Sigma, F1804, 1:5000), and anti-β-actin (Sigma, A2228, 1:4000) antibodies.

### Chromatin Immunoprecipitation

Chromatin Immunoprecipitation (ChIP) assays were performed essentially as described before (Wang et al., 2020; Liu et al., 2021a). In brief, chromatin in control and treated cells were cross-linked with 1% formaldehyde. Cells were incubated in lysis buffer (150 mM NaCl, 25 mM Tris pH 7.5, 1% Triton X-100, 0.1% SDS, 0.5% deoxycholate) supplemented with protease inhibitor tablet and PMSF. DNA was fragmented into ∼200 bp pieces using a Branson 250 sonicator. Aliquots of lysates containing 200 μg of protein were used for each immunoprecipitation reaction with anti-Twist1 (Proteintech, 25465-1), anti-Slug (Cell Signaling Technology, 9585), anti-Zeb1 (Cell Signaling Technology, 3396), anti-Snail (Cell Signaling Technology, 3879), anti-anti-acetyl H3 (Millipore, 06-599), anti-acetyl H4 (Millipore, 06-598), anti-HDAC1 (Santa Cruz, sc-7872), or pre-immune IgG. For re-ChIP, immune complexes were eluted with the elution buffer (1% SDS, 100 mM NaCO_3_), diluted with the re-ChIP buffer (1% Triton X-100, 2 mM EDTA, 150 mM NaCl, 20 mM Tris pH 8.1), and subject to immunoprecipitation with a second antibody of interest.

### RNA Isolation and Real-Time PCR

RNA was extracted with the RNeasy RNA isolation kit (Qiagen). Reverse transcriptase reactions were performed using a SuperScript First-strand Synthesis System (Invitrogen) as previously described (Dong et al., 2020; Hong et al., 2020; Wu T. et al., 2020; Yang et al., 2020a,b, 2021; Zhang et al., 2020; Kong et al., 2021b). Real-time PCR reactions were performed on an ABI Prism 7500 system. Ct values of target genes were normalized to the Ct values of housekeeping control gene (18s rRNA, 5′-CGCGGTTCTATTTTGTTGGT-3′ and 5′-TCGTCTTCGAAACTCCGACT-3′ for both human and mouse genes) using the ΔΔCt method and expressed as relative mRNA expression levels compared to the control group which is arbitrarily set as 1.

### 5-Ethynyl-2′-Deoxyuridine Incorporation Assay

5-ethynyl-2′-deoxyuridine (EdU) incorporation assay was performed in triplicate wells with a commercially available kit (Thermo Fisher Scientific) per vendor instruction. Briefly, the EdU solution was diluted with the culture media and added to the cells for an incubation period of 2 h at 37°C. After several washes with 1XPBS, the cells were then fixed with 4% formaldehyde and stained with Alexa Fluor^TM^ 488. The nucleus was counter-stained with DAPI. The images were visualized by fluorescence microscopy and analyzed with Image-Pro Plus (Media Cybernetics). For each group, at least six different fields were randomly chosen and the positively stained cells were counted and divided by the number of total cells. The data are expressed as relative EdU staining compared to the control group arbitrarily set as 1.

### Statistical Analysis

One-way ANOVA with *post hoc* Scheff’e analyses were performed by SPSS software (IBM SPSS v18.0, Chicago, IL, United States). Unless otherwise specified, values of *p* < 0.05 were considered statistically significant.

## Results

### Chloride Channel Accessory 2 Expression Is Down-Regulated in Activated Cardiac Fibroblasts

**FIGURE 1:**
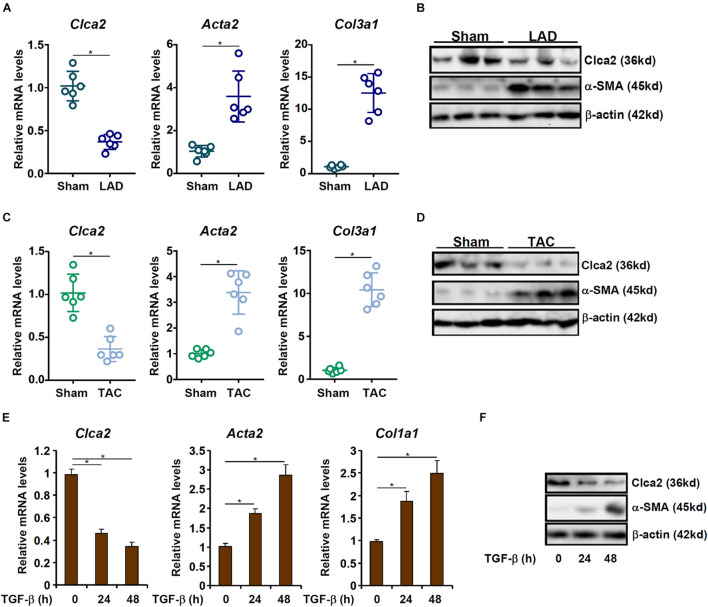
Chloride channel accessory 2 (Clca2) expression is down-regulated in activated cardiac fibroblasts. **(A,B)** C57B/6 mice were subjected to the LAD procedure to induce myocardial infarction. The mice were sacrificed 7 day after the surgery and primary cardiac fibroblasts were isolated. Clca2 expression was examined by qPCR and Western. *N* = 6 mice for each group. **(C,D)** C57B/6 mice were subjected to the TAC procedure to induce myocardial hypertrophy. The mice were sacrificed 7 day after the surgery and primary cardiac fibroblasts were isolated. Clca2 expression was examined by qPCR and Western. *N* = 6 mice for each group. **(E,F)** Primary cardiac fibroblasts were isolated from C57B/6 mice and treated with TGF-β (2 ng/ml). Cells were harvested at indicated time points and Clca2 expression was examined by qPCR and Western. Error bars represent SD (*p < 0.05, two-way Student’s *t*-test). All experiments were repeated three times and one representative experiment is shown.

When exposed to injurious stimuli, cardiac resident fibroblasts undergo *trans-*differentiation and become mature myofibroblasts to mediate the fibrogenic response. In order to compare Clca2 expression in quiescent cardiac fibroblasts and activated cardiac fibroblasts, C57B/6 mice were subjected to the LAD procedure to induce myocardial infarction; previous investigations have shown that FMyT peaks at 7 day after the surgery (Kanisicak et al., 2016). It was observed that compared to the sham-operated mice, expression levels of Acta2 (encoding α-SMA) and Col1a1 (encoding collagen type I), two typical myofibroblast markers, were significantly up-regulated in the primary cardiac fibroblasts isolated from the LAD-operated mice; on the contrary, Clca2 expression was down-regulated in the activated cardiac fibroblasts compared to the quiescent cardiac fibroblasts (Figure 1A). Western blotting confirmed that Clca2 protein levels were down-regulated as well (Figure 1B). In the second model of myocardial fibrosis, C57B/6 mice were subjected to the TAC procedure; FMyT typically peaks at 7 day after the surgery (Bursac, 2014). QPCR (Figure 1C) and Western blotting (Figure 1D) showed that Clca2 expression was lower in the activated cardiac fibroblasts isolated from the TAC mice than in the quiescent cardiac fibroblasts isolated from the sham mice, opposite to the changes in Acta2 expression and Col1a1 expression.

TGF-β is one of most potent inducer of FMyT and myocardial fibrosis (Davis and Molkentin, 2014). When quiescent cardiac fibroblasts were treated with TGF-β, it was found that both Acta2 and Col1a1 were progressively up-regulated whereas Clca2 expression was concomitantly down-regulated (Figures 1E,F).

### TWIST1 Mediates Chloride Channel Accessory 2 *Trans-*Repression in Cardiac Fibroblasts

**FIGURE 2:**
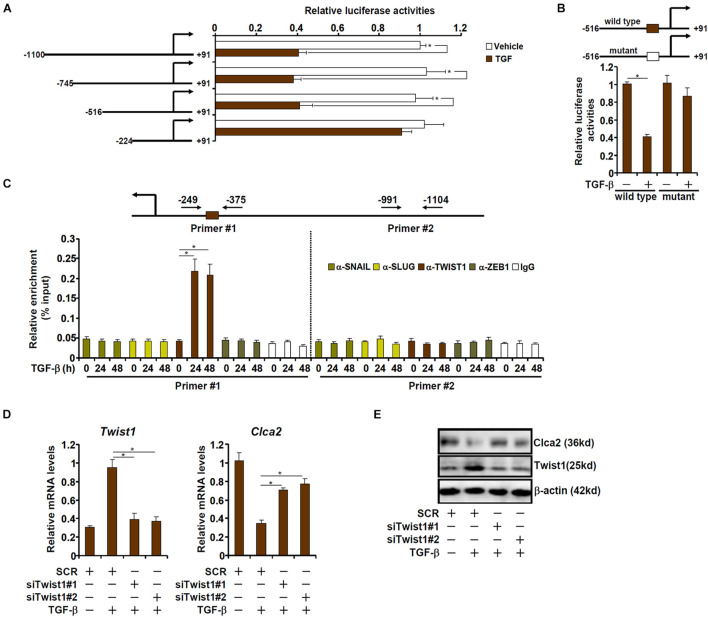
TWIST1 mediates Clca2 *trans-*repression in cardiac fibroblasts. **(A)** Different Clca2 promoter-luciferase constructs were transfected into mouse embryonic fibroblasts (MEFs) followed by treatment with TGF-β. Luciferase activities were normalized by protein concentration and GFP fluorescence. **(B)** Wild type and mutant Clca2 promoter-luciferase constructs were transfected into MEFs followed by treatment with TGF-β. Luciferase activities were normalized by protein concentration and GFP fluorescence. **(C)** Primary cardiac fibroblasts were treated with TGF-β (2 ng/ml) and were harvested at indicated time points. ChIP assays were performed with indicated antibodies. **(D,E)** Primary cardiac fibroblasts were transfected with indicated siRNAs followed by treatment with TGF-β (2 ng/ml). Gene expression was examined by qPCR and Western. Error bars represent SD (*p < 0.05, two-way Student’s *t*-test). All experiments were repeated three times and one representative experiment is shown.

We next determined whether down-regulation of Clca2 expression by TGF-β occurred at the transcriptional level. A series of Clca2 promoter-luciferase reporter constructs were transfected into mouse embryonic fibroblasts (MEFs) followed by TGF-β treatment. As shown in Figure 2A, TGF-β treatment decreased the activity of the full-length Clca2 promoter (−1100/ + 91) suggesting that TGF-β could indeed repress Clca2 transcription. However, when deletions introduced to the full-length Clca2 promoter extended beyond −516, TGF-β treatment could no longer repress the Clca2 promoter activity. A closer examination revealed a conserved E-box (CAGGTG) located between −516 and −224 of the Clca2 promoter; mutation of the E-box completely abrogated the response to TGF-β treatment (Figure 2B).

The E-box binding family of zinc finger transcription repressors include Snail, Slug, Twist1, and Zeb1 (Kalluri and Weinberg, 2009). ChIP assay was performed to determine which one of these transcription factors (TFs). As shown in Figure 2C, Twist1, but not Snail, Slug, or Zeb1, occupied the proximal Clca2 promoter containing the E-box in response to TGF-β treatment; none of the TFs were detected on the distal Clca2 promoter. To further validate the role of Twist1 in Clca2 *trans-*repression, endogenous Twist1 was depleted with two independent pairs of siRNAs. Twist1 knockdown partially restored Clca2 expression in the presence of TGF-β in cardiac fibroblasts (Figures 2D,E).

### TWIST1 Represses Chloride Channel Accessory 2 Transcription by Promoting Histone Deacetylation

**FIGURE 3:**
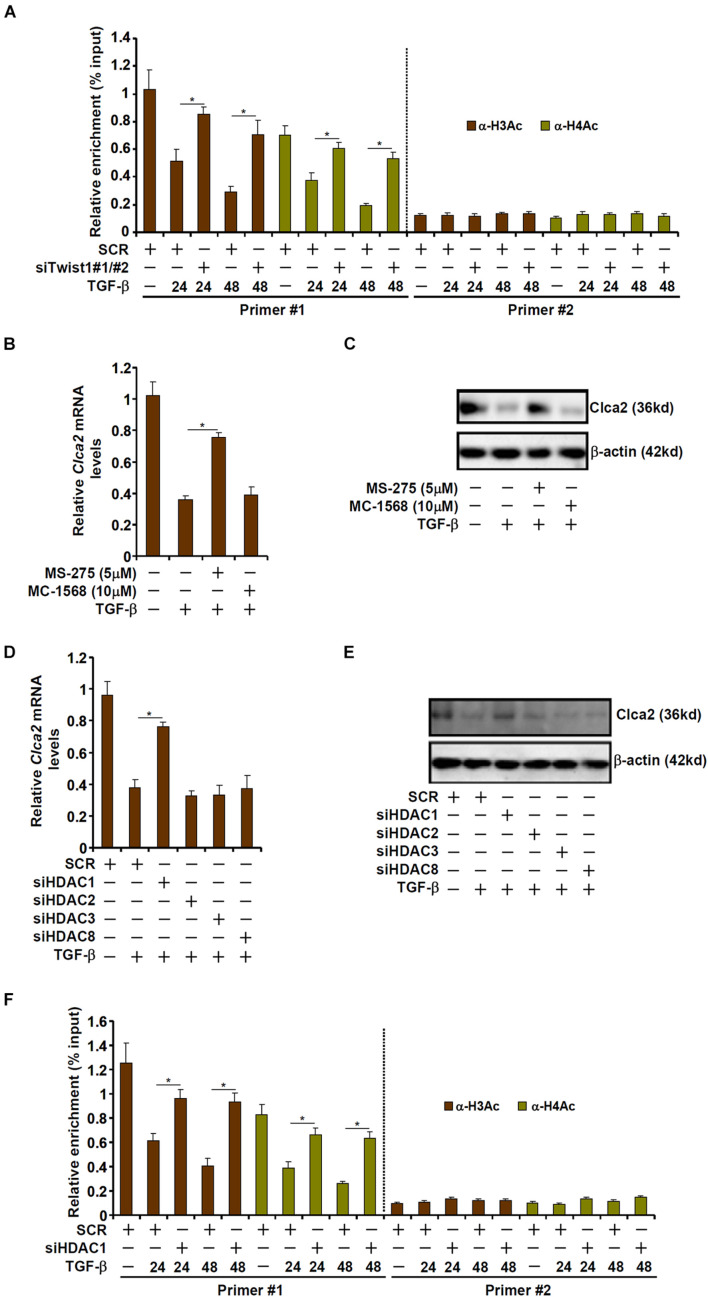
TWIST1 represses Clca2 transcription by promoting histone deacetylation. **(A)** Primary murine cardiac fibroblasts were transfected with indicated siRNAs by treatment with TGF-β (2 ng/ml). ChIP assays were performed with indicated antibodies. **(B,C)** Primary murine cardiac fibroblasts were treated with TGF-β (2 ng/ml) in the presence or absence of different HDAC inhibitors. Clca2 expression was examined by qPCR and Western. **(D,E)** Primary murine cardiac fibroblasts were transfected with indicated siRNAs by treatment with TGF-β (2 ng/ml). Clca2 expression was examined by qPCR and Western. **(F)** Primary cardiac murine fibroblasts were transfected with indicated siRNAs by treatment with TGF-β (2 ng/ml). ChIP assays were performed with indicated antibodies. Error bars represent SD (*p < 0.05, two-way Student’s *t*-test). All experiments were repeated three times and one representative experiment is shown.

Transcriptional repression is usually associated with erasure of histone acetylation surrounding the promoter region (Jenuwein and Allis, 2001). As shown in Figure 3A, TGF-β treatment led to disappearance of acetylated histone H3 and acetylated histone H4 from the proximal, but not the distal, Clca2 promoter; Twist1 knockdown normalized histone acetylation, suggesting that histone deacetylases (HDACs) might be involved in Twist1 mediated Clca2 *trans-*repression. HDACs can be categorized into three classes: class I and class II HDACs primarily catalyze histone deacetylation whereas class III HDACs (the sirtuins) primarily catalyze non-histone lysine deacetylation (Yang and Seto, 2008). Pre-treatment with a pan-class I HDAC inhibitor (MS-275), but not a pan-class II HDAC inhibitor (MC-1568), blocked TGF-β induced Clca2 repression (Figures 3B,C), indicating that class I HDAC might be involved in Clca2 *trans-*repression.

Class I HDACs include HDAC1, HDAC2, HDAC3, and HDAC8. When individual class I HDACs were depleted with siRNAs, it was discovered that only HDAC1 knockdown significantly attenuated Clca2 repression by TGF-β treatment (Figures 3D,E). Consistently, HDAC1 knockdown largely normalized histone acetylation levels surrounding the Clca2 promoter (Figure 3F).

### TWIST1 Interacts With and Recruits HDAC1 to Repress Chloride Channel Accessory 2 Transcription

**FIGURE 4:**
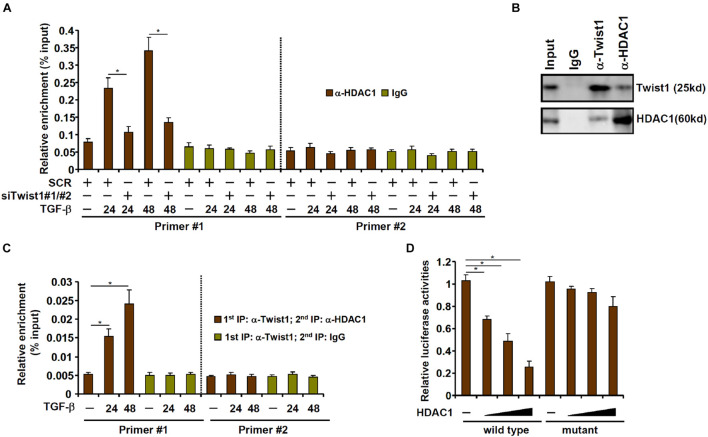
TWIST1 interacts with and recruits HDAC1 to repress Clca2 transcription. **(A)** Primary cardiac murine fibroblasts were transfected with indicated siRNAs by treatment with TGF-β (2 ng/ml). ChIP assays were performed with indicated antibodies. **(B)** Immunoprecipitation was performed with whole cell lysates from primary murine cardiac fibroblasts. **(C)** Primary murine cardiac fibroblasts were treated with or without TGF-β (2 ng/ml) for 48 h. Re-ChIP was performed with indicated antibodies. **(D)** Wild type and mutant Clca2 promoter-luciferase constructs were transfected into MEFs with increasing doses of HDAC1 followed by treatment with TGF-β. Luciferase activities were normalized by protein concentration and GFP fluorescence. Error bars represent SD (*p < 0.05, two-way Student’s *t*-test). All experiments were repeated three times and one representative experiment is shown.

We next investigated the possibility that Twist1 recruits HDAC1 to repress Clca2 transcription. ChIP assay showed that occupancies of HDAC1 on the Clca2 promoter were enhanced following TGF-β treatment with a kinetics similar to Twist1; Twist1 depletion blocked HDAC1 binding to the Clca2 promoter (Figure 4A). Co-immunoprecipitation confirmed that Twist1 and HDAC1 could interact with each other in cardiac fibroblasts (Figure 4B). Importantly, Re-ChIP assay showed that the Twist1-HDAC1 interaction was significantly cemented by TGF-β treatment on the Clca2 promoter (Figure 4C). In addition, whereas HDAC1 over-expression dose-dependently repressed the Clca2 promoter activity in reporter assay the mutant Clca2 promoter without the intact E-box was completely refractory to HDAC1 over-expression (Figure 4D).

### Chloride Channel Accessory 2 Regulates Activation of Cardiac Fibroblasts

**FIGURE 5:**
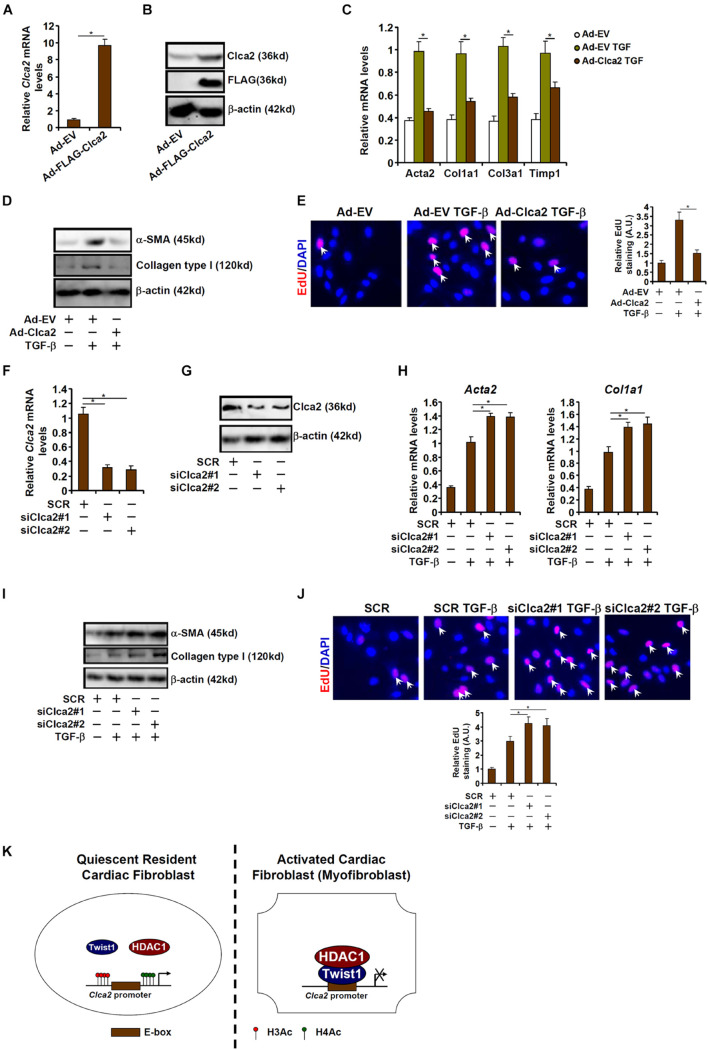
Chloride channel accessory 2 (Clca2) regulates activation of cardiac fibroblasts. **(A–E)** Primary murine cardiac fibroblasts were infected with adenovirus carrying a Clca2 expression vector (Ad-FLAG-Clca2) or an empty vector (Ad-EV) followed by treatment with TGF-β. Expression levels of Clca2 were examined by qPCR **(A)** and Western **(B)**. Pro-fibrogenic genes were examined by qPCR **(C)** and Western **(D)**. Cell proliferation was examined by EdU incorporation **(E)**. **(F–J)** Primary murine cardiac fibroblasts were transfected with indicated siRNAs followed by treatment with TGF-β. Expression levels of Clca2 were examined by qPCR **(F)** and Western **(G)**. Pro-fibrogenic genes were examined by qPCR **(H)** and Western **(I)**. Cell proliferation was examined by EdU incorporation **(J)**. Error bars represent SD (*p < 0.05, two-way Student’s *t*-test). All experiments were repeated three times and one representative experiment is shown. **(K)** A schematic model.

Finally, an attempt was made to place the finding that Clca2 transcription was epigenetically repressed during cardiac fibroblast activation in a pathophysiological perspective. To this end, primary murine cardiac fibroblasts were transduced with adenovirus carrying a Clca2 expression vector (Ad-FLAG-Clca2) or an empty vector (Ad-EV). Ad-FLAG-Clca2 transduction significantly boosted Clca2 expression in cardiac fibroblasts (Figures 5A,B). More important, Clca2 over-expression significantly down-regulated the expression of myofibroblast marker genes at both mRNA (Figure 5C) and protein (Figure 5D) levels. In addition, Clca2 over-expression attenuated proliferation of cardiac fibroblasts as measured by EdU incorporation (Figure 5E).

Alternatively, Clca2 expression was depleted with two separate pairs of siRNAs (Figures 5F,G). Clca2 knockdown further enhanced TGF-β induced expression of myofibroblast marker genes (Figures 5H,I) and augmented cell proliferation (Figure 5J). Together, these data suggest that Clca2 might regulate FMyT *in vitro*.

## Discussion

Recent investigations have provided irrefutable evidence to support resident fibroblasts as the primary source of myofibroblasts contributing to cardiac fibrosis (Travers et al., 2016). Dynamic transcriptomic changes highlight the transition from quiescent cardiac fibroblasts to mature myofibroblasts (Krstevski et al., 2020). We show here that Twist1 is responsible for Clca2 *trans-*repression in activated cardiac fibroblasts by directly binding to the E-box element located on the Clca2 promoter (Figure 5K). Consistent with our observation, Al-Hattab et al. (2018) have previously reported that Twist1 transcription can be activated by TGF-β in cardiac fibroblasts, which is mediated by scleraxis. Of note, several studies have found that Twist1 can be placed among signature markers for cardiac fibroblasts (Zhou et al., 2010; Liu et al., 2016; Han et al., 2021). Whether or not Twist1 can directly regulate cardiac fibrosis remains to be determined. On the one hand, pharmaceutical inhibition and fibroblast-specific deletion of Twist1 have been shown to mitigate liver fibrosis (Dong et al., 2020) and skin fibrosis (Palumbo-Zerr et al., 2017), respectively, supporting Twist1 as a promoter of tissue fibrosis. On the other hand, Twist1 residing in the parenchymal cells, the mesenchymal cells, or infiltrating immune cells is able to rein in tissue injury and antagonize tissue fibrosis (Tan et al., 2017; Ren et al., 2019, 2021). Therefore, more studies should be conducted to test the feasibility of targeting Twist1 as a therapeutic strategy against aberrant cardiac fibrosis.

Our data indicate that Twist1 represses Clca2 transcription *via*, at least in part, by recruiting the histone deacetylase HDAC1. Curiously, our observation is in contrast to a previous study by Xu et al. (2006) where it was demonstrated that HDAC1, recruited by the RFX1, directly binds to the collagen type I promoter (Col1a2) and represses Col1a2 transcription in lung fibroblasts in response to IFN-γ treatment. It is likely that HDAC1 may exert differential effects on tissue fibrogenesis depending on the stimuli and the cell type. Global deletion of HDAC1 in mice results in early developmental arrest and embryonic lethality precluding the analysis of cardiac fibrosis in adult animals (Montgomery et al., 2007). More recently, Renaud et al. (2015) have shown that administration of a pan-HDAC inhibitor (HDACi) attenuates cardiac fibrosis in mice subjected to pressure overload although the mechanism is less clear but possibly can be attributable to HDAC1-mediated repression of miR-133a, an anti-fibrotic non-regulatory RNA. Of note, HDAC1-null MEFs display weakened proliferation compared to wild type MEFs (Yamaguchi et al., 2010), suggesting that HDAC1 deficiency may prevent cardiac fibrosis by limiting the expansion of myofibroblasts *in vivo* (Yamaguchi et al., 2010). Future studies employing fibroblast/myofibroblast conditional transgenic animals should clarify the role of HDAC1 in cardiac fibrosis.

We present data to show that manipulating Clca2 expression in cardiac fibroblasts influences FMyT *in vitro*. The underlying mechanism, however, awaits further investigation. Previous studies have shown that a variety of chloride channels may contribute to myofibroblast maturation *via* the MAPK-p38 signaling pathway (Shukla et al., 2014) or the PI3K-Akt signaling pathway (Sun et al., 2016) or the PKC signaling pathway (El Chemaly et al., 2014). Alternatively, chloride intracellular channel 4 (CLIC4) has been shown to promote TGF-β induced FMyT by inducing a dominant negative SMAD7 splicing isoform (Shukla et al., 2016). Despite the fact that several studies have provided evidence to show that chloride channel inhibitors/blockers can potentially attenuate the activation of cardiac fibroblasts (El Chemaly et al., 2014; Tian et al., 2018; Chen P. H. et al., 2021), no consensus seems to exist regarding the underlying mechanisms. It is therefore imperative for future investigators to focus on delineating the mode of action for Clca2 in the process of FMyT so that the plethora of data, including the ones presented here, can be exploited in the development of novel therapeutic solutions to treat adverse cardiac remodeling and heart failure.

## Data Availability Statement

The original contributions presented in the study are included in the article/supplementary material, further inquiries can be directed to the corresponding author/s.

## Ethics Statement

The animal study was reviewed and approved by Ethics Committee on Humane Treatment of Laboratory Animals of Jiangsu Health Vocational College.

## Author Contributions

MF conceived the project and secured funding and provided supervision. TS, YX, and MF designed the experiments. TS and YX performed the experiments, collected the data, and analyzed the data. All authors contributed to the drafting and editing of manuscript.

## Conflict of Interest

The authors declare that the research was conducted in the absence of any commercial or financial relationships that could be construed as a potential conflict of interest.

## Publisher’s Note

All claims expressed in this article are solely those of the authors and do not necessarily represent those of their affiliated organizations, or those of the publisher, the editors and the reviewers. Any product that may be evaluated in this article, or claim that may be made by its manufacturer, is not guaranteed or endorsed by the publisher.

## References

[B1] Al-Hattab D. S. Safi H. A. Nagalingam R. S. Bagchi R. A. Stecy M. T. Czubryt M. P. (2018). Scleraxis regulates Twist1 and Snai1 expression in the epithelial-to-mesenchymal transition. *Am. J. Physiol. Heart Circ. Physiol.* 315 H658–H668. 10.1152/ajpheart.00092.2018 29906225

[B2] Angrisani A. Di Fiore A. Di Trani C. A. Fonte S. Petroni M. Lospinoso Severini L. (2021). Specific Protein 1 and p53 interplay modulates the expression of the KCTD-containing Cullin3 adaptor suppressor of Hedgehog 2. *Front. Cell Dev. Biol.* 9:638508. 10.3389/fcell.2021.638508 33898425 PMC8060498

[B3] Bursac N. (2014). Cardiac fibroblasts in pressure overload hypertrophy: the enemy within? *J. Clin. Invest.* 124 2850–2853. 10.1172/JCI76628 24937423 PMC4071394

[B4] Chen B. Fan Z. Sun L. Chen J. Feng Y. Fan X. (2020a). Epigenetic activation of the small GTPase TCL contributes to colorectal cancer cell migration and invasion. *Oncogenesis* 9:86. 10.1038/s41389-020-00269-9 32999272 PMC7528090

[B5] Chen B. Yuan Y. Sun L. Chen J. Yang M. Yin Y. (2020b). MKL1 mediates TGF-β Induced RhoJ transcription to promote breast cancer cell migration and invasion. *Front. Cell Dev. Biol.* 8:832. 10.3389/fcell.2020.00832 32984327 PMC7478007

[B6] Chen B. Zhao Q. Xu T. Yu L. Zhuo L. Yang Y. (2020c). BRG1 activates PR65A transcription to regulate NO bioavailability in vascular endothelial cell. *Front. Cell Dev. Biol.* 8:774. 10.3389/fcell.2020.00774 32903816 PMC7443572

[B7] Chen B. Zhu Y. Chen J. Feng Y. Xu Y. (2021). Activation of TCL transcription by lysine demethylase KDM4B in colorectal cancer cells. *Front. Cell Dev. Biol.* 9:617549. 10.3389/fcell.2021.617549 34249900 PMC8260841

[B8] Chen P. H. Chung C. C. Lin Y. F. Kao Y. H. Chen Y. J. (2021). Lithium reduces migration and collagen synthesis activity in human cardiac fibroblasts by inhibiting store-operated Ca(2+) entry. *Int. J. Mol. Sci.* 22:842. 10.3390/ijms22020842 33467715 PMC7830715

[B9] Davis J. Molkentin J. D. (2014). Myofibroblasts: trust your heart and let fate decide. *J. Mol. Cell Cardiol.* 70 9–18. 10.1016/j.yjmcc.2013.10.019 24189039 PMC3995855

[B10] Dong W. Kong M. Zhu Y. Shao Y. Wu D. Lu J. (2020). Activation of TWIST transcription by chromatin remodeling Protein BRG1 contributes to liver fibrosis in mice. *Front. Cell Dev. Biol.* 8:340. 10.3389/fcell.2020.00340 32478075 PMC7237740

[B11] Dong W. Zhu Y. Zhang Y. Fan Z. Zhang Z. Fan X. (2021). BRG1 Links TLR4 trans-activation to LPS-Induced SREBP1a expression and liver injury. *Front. Cell Dev. Biol.* 9:617073. 10.3389/fcell.2021.617073 33816466 PMC8012493

[B12] El Chemaly A. Norez C. Magaud C. Bescond J. Chatelier A. Fares N. (2014). ANO1 contributes to angiotensin-II-activated Ca2+-dependent Cl- current in human atrial fibroblasts. *J. Mol. Cell Cardiol.* 68 12–19. 10.1016/j.yjmcc.2013.12.027 24412532

[B13] Gao Q. Y. Zhang H. F. Tao J. Chen Z. T. Liu C. Y. Liu W. H. (2020). Mitochondrial fission and mitophagy reciprocally orchestrate cardiac fibroblasts activation. *Front. Cell Dev. Biol.* 8:629397. 10.3389/fcell.2020.629397 33585469 PMC7874126

[B14] Gonzalez A. Schelbert E. B. Diez J. Butler J. (2018). myocardial interstitial fibrosis in heart failure: biological and translational perspectives. *J. Am. Coll. Cardiol.* 71 1696–1706. 10.1016/j.jacc.2018.02.021 29650126

[B15] Gruber A. D. Schreur K. D. Ji H. L. Fuller C. M. Pauli B. U. (1999). Molecular cloning and transmembrane structure of hCLCA2 from human lung, trachea, and mammary gland. *Am. J. Physiol.* 276 C1261–C1270. 10.1152/ajpcell.1999.276.6.C1261 10362588

[B16] Han J. K. Shin Y. Sohn M. H. Choi S. B. Shin D. You Y. (2021). Direct conversion of adult human fibroblasts into functional endothelial cells using defined factors. *Biomaterials* 272:120781. 10.1016/j.biomaterials.2021.120781 33848809

[B17] He S. Lu Y. Guo Y. Li S. Lu X. Shao S. (2021). Kruppel-Like Factor 15 modulates CXCL1/CXCR2 signaling-mediated inflammatory response contributing to angiotensin II-induced cardiac remodeling. *Front. Cell Dev. Biol.* 9:644954. 10.3389/fcell.2021.644954 33869197 PMC8047332

[B18] Hong W. Kong M. Qi M. Bai H. Fan Z. Zhang Z. (2020). BRG1 mediates nephronectin activation in hepatocytes to Promote T lymphocyte infiltration in ConA-Induced hepatitis. *Front. Cell Dev. Biol.* 8:587502. 10.3389/fcell.2020.587502 33553140 PMC7858674

[B19] Jentsch T. J. Pusch M. (2018). CLC chloride channels and transporters: structure. Function, physiology, and disease. *Physiol. Rev.* 98 1493–1590. 10.1152/physrev.00047.2017 29845874

[B20] Jenuwein T. Allis C. D. (2001). Translating the histone code. *Science* 293 1074–1080. 10.1126/science.1063127 11498575

[B21] Kalluri R. Weinberg R. A. (2009). The basics of epithelial-mesenchymal transition. *J. Clin. Invest.* 119 1420–1428. 10.1172/JCI39104 19487818 PMC2689101

[B22] Kanisicak O. Khalil H. Ivey M. J. Karch J. Maliken B. D. Correll R. N. (2016). Genetic lineage tracing defines myofibroblast origin and function in the injured heart. *Nat. Commun.* 7:12260. 10.1038/ncomms12260 27447449 PMC5512625

[B23] Kaur H. Takefuji M. Ngai C. Y. Carvalho J. Bayer J. Wietelmann A. (2016). Targeted ablation of periostin-expressing activated fibroblasts prevents adverse cardiac remodeling in mice. *Circ. Res.* 118 1906–1917. 10.1161/CIRCRESAHA.116.308643 27140435

[B24] Kong M. Dong W. Xu H. Fan Z. Miao X. Guo Y. (2021a). Choline kinase alpha is a novel transcriptional target of the brg1 in hepatocyte: implication in liver regeneration. *Front. Cell Dev. Biol.* 9:705302. 10.3389/fcell.2021.705302 34422825 PMC8377418

[B25] Kong M. Dong W. Zhu Y. Fan Z. Miao X. Guo Y. (2021b). Redox-sensitive activation of CCL7 by BRG1 in hepatocytes during liver injury. *Redox Biol.* 46:102079. 10.1016/j.redox.2021.102079 34454163 PMC8406035

[B26] Kong M. Zhu Y. Shao J. Fan Z. Xu Y. (2021c). The chromatin remodeling protein BRG1 Regulates SREBP maturation by activating SCAP transcription in hepatocytes. *Front. Cell Dev. Biol.* 9:622866. 10.3389/fcell.2021.622866 33718362 PMC7947303

[B27] Kramann R. Schneider R. K. DiRocco D. P. Machado F. Fleig S. Bondzie P. A. (2015). Perivascular Gli1+ progenitors are key contributors to injury-induced organ fibrosis. *Cell Stem Cell* 16 51–66. 10.1016/j.stem.2014.11.004 25465115 PMC4289444

[B28] Krstevski C. Cohen C. D. Dona M. S. I. Pinto A. R. (2020). New perspectives of the cardiac cellular landscape: mapping cellular mediators of cardiac fibrosis using single-cell transcriptomics. *Biochem. Soc. Trans.* 48 2483–2493. 10.1042/BST20191255 33259583

[B29] Liu C. Han Y. Gu X. Li M. Du Y. Feng N. (2021a). Paeonol promotes Opa1-mediated mitochondrial fusion via activating the CK2alpha-Stat3 pathway in diabetic cardiomyopathy. *Redox Biol.* 46:102098. 10.1016/j.redox.2021.102098 34418601 PMC8385203

[B30] Liu L. Zhao Q. Kong M. Mao L. Yang Y. Xu Y. (2021b). Myocardin-related transcription factor A (MRTF-A) regulates integrin beta 2 transcription to promote macrophage infiltration and cardiac hypertrophy in mice. *Cardiovasc. Res.* cvab110. 10.1093/cvr/cvab110 33752236

[B31] Liu L. Zhao Q. Lin L. Yang G. Yu L. Zhuo L. (2021c). Myeloid MKL1 disseminates cues to promote cardiac hypertrophy in mice. *Front. Cell Dev. Biol.* 9:583492. 10.3389/fcell.2021.583492 33898415 PMC8063155

[B32] Liu C. Hu T. Cai Z. Xie Q. Yuan Y. Li N. (2020). Nucleotide-binding oligomerization domain-like receptor 3 deficiency attenuated isoproterenol-induced cardiac fibrosis via reactive oxygen species/high mobility group Box 1 Protein axis. *Front. Cell Dev. Biol.* 8:713. 10.3389/fcell.2020.00713 32850832 PMC7431462

[B33] Liu Z. Chen O. Zheng M. Wang L. Zhou Y. Yin C. (2016). Re-patterning of H3K27me3, H3K4me3 and DNA methylation during fibroblast conversion into induced cardiomyocytes. *Stem Cell Res.* 16 507–518. 10.1016/j.scr.2016.02.037 26957038 PMC4828257

[B34] Mollmann H. Nef H. M. Kostin S. von Kalle C. Pilz I. Weber M. (2006). Bone marrow-derived cells contribute to infarct remodelling. *Cardiovasc. Res.* 71 661–671. 10.1016/j.cardiores.2006.06.013 16854401

[B35] Montgomery R. L. Davis C. A. Potthoff M. J. Haberland M. Fielitz J. Qi X. (2007). Histone deacetylases 1 and 2 redundantly regulate cardiac morphogenesis, growth, and contractility. *Genes Dev.* 21 1790–1802. 10.1101/gad.1563807 17639084 PMC1920173

[B36] Moore-Morris T. Guimaraes-Camboa N. Banerjee I. Zambon A. C. Kisseleva T. Velayoudon A. (2014). Resident fibroblast lineages mediate pressure overload-induced cardiac fibrosis. *J. Clin. Invest.* 124 2921–2934. 10.1172/JCI74783 24937432 PMC4071409

[B37] Palumbo-Zerr K. Soare A. Zerr P. Liebl A. Mancuso R. Tomcik M. (2017). Composition of TWIST1 dimers regulates fibroblast activation and tissue fibrosis. *Ann. Rheum Dis.* 76 244–251. 10.1136/annrheumdis-2015-208470 27113414

[B38] Ramena G. Yin Y. Yu Y. Walia V. Elble R. C. (2016). CLCA2 Interactor EVA1 is required for mammary epithelial cell differentiation. *PLoS One* 11:e0147489. 10.1371/journal.pone.0147489 26930581 PMC4773014

[B39] Ren J. Xu Y. Lu X. Wang L. Ide S. Hall G. (2021). Twist1 in podocytes ameliorates podocyte injury and proteinuria by limiting CCL2-dependent macrophage infiltration. *JCI Insight* 6:e148109. 10.1172/jci.insight.148109 34369383 PMC8410065

[B40] Ren J. Zhang J. Rudemiller N. P. Griffiths R. Wen Y. Lu X. (2019). Twist1 in infiltrating macrophages attenuates kidney fibrosis via matrix metallopeptidase 13-Mediated matrix degradation. *J. Am. Soc. Nephrol.* 30 1674–1685. 10.1681/ASN.2018121253 31315922 PMC6727252

[B41] Renaud L. Harris L. G. Mani S. K. Kasiganesan H. Chou J. C. Baicu C. F. (2015). HDACs regulate miR-133a expression in pressure overload-induced cardiac fibrosis. *Circ. Heart Fail.* 8 1094–1104. 10.1161/CIRCHEARTFAILURE.114.001781 26371176 PMC4651803

[B42] Sasaki Y. Koyama R. Maruyama R. Hirano T. Tamura M. Sugisaka J. (2012). CLCA2, a target of the p53 family, negatively regulates cancer cell migration and invasion. *Cancer Biol. Ther.* 13 1512–1521. 10.4161/cbt.22280 22990203 PMC3542243

[B43] Seltmann K. Meyer M. Sulcova J. Kockmann T. Wehkamp U. Weidinger S. (2018). Humidity-regulated CLCA2 protects the epidermis from hyperosmotic stress. *Sci. Transl. Med.* 10:eaao4650. 10.1126/scitranslmed.aao4650 29743348

[B44] Shukla A. Edwards R. Yang Y. Hahn A. Folkers K. Ding J. (2014). CLIC4 regulates TGF-beta-dependent myofibroblast differentiation to produce a cancer stroma. *Oncogene* 33 842–850. 10.1038/onc.2013.18 23416981 PMC3912213

[B45] Shukla A. Yang Y. Madanikia S. Ho Y. Li M. Sanchez V. (2016). Elevating CLIC4 in multiple cell types reveals a TGF- dependent induction of a dominant negative Smad7 splice variant. *PLoS One* 11:e0161410. 10.1371/journal.pone.0161410 27536941 PMC4990216

[B46] Stempien-Otero A. Kim D. H. Davis J. (2016). Molecular networks underlying myofibroblast fate and fibrosis. *J. Mol. Cell Cardiol.* 97 153–161. 10.1016/j.yjmcc.2016.05.002 27167848 PMC5482716

[B47] Sun L. Dong Y. Zhao J. Yin Y. Zheng Y. (2016). The CLC-2 chloride channel modulates ECM synthesis, differentiation, and migration of human conjunctival fibroblasts via the PI3K/Akt signaling pathway. *Int. J. Mol. Sci.* 17:910. 10.3390/ijms17060910 27294913 PMC4926444

[B48] Surguchov A. Surgucheva I. Sharma M. Sharma R. Singh V. (2017). Pore-forming proteins as mediators of novel epigenetic mechanism of epilepsy. *Front. Neurol.* 8:3. 10.3389/fneur.2017.00003 28149289 PMC5241277

[B49] Talman V. Ruskoaho H. (2016). Cardiac fibrosis in myocardial infarction-from repair and remodeling to regeneration. *Cell Tissue Res.* 365 563–581. 10.1007/s00441-016-2431-9 27324127 PMC5010608

[B50] Tan J. Tedrow J. R. Nouraie M. Dutta J. A. Miller D. T. Li X. (2017). Loss of Twist1 in the mesenchymal compartment promotes increased fibrosis in experimental lung injury by enhanced expression of CXCL12. *J. Immunol.* 198 2269–2285. 10.4049/jimmunol.1600610 28179498 PMC5337810

[B51] Tian X. Q. Ma K. T. Wang X. W. Wang Y. Guo Z. K. Si J. Q. (2018). Effects of the calcium-activated chloride channel inhibitors T16Ainh-A01 and CaCCinh-A01 on cardiac fibroblast function. *Cell Physiol. Biochem.* 49 706–716. 10.1159/000493036 30165368

[B52] Tomasek J. J. Gabbiani G. Hinz B. Chaponnier C. Brown R. A. (2002). Myofibroblasts and mechano-regulation of connective tissue remodelling. *Nat. Rev. Mol. Cell Biol.* 3 349–363. 10.1038/nrm809 11988769

[B53] Travers J. G. Kamal F. A. Robbins J. Yutzey K. E. Blaxall B. C. (2016). Cardiac fibrosis: the fibroblast awakens. *Circ. Res.* 118 1021–1040. 10.1161/CIRCRESAHA.115.306565 26987915 PMC4800485

[B54] van den Borne S. W. Diez J. Blankesteijn W. M. Verjans J. Hofstra L. Narula J. (2010). Myocardial remodeling after infarction: the role of myofibroblasts. *Nat. Rev. Cardiol.* 7 30–37. 10.1038/nrcardio.2009.199 19949426

[B55] Walia V. Ding M. Kumar S. Nie D. Premkumar L. S. Elble R. C. (2009). hCLCA2 Is a p53-inducible inhibitor of breast cancer cell proliferation. *Cancer Res.* 69 6624–6632. 10.1158/0008-5472.CAN-08-4101 19654313 PMC2745301

[B56] Walia V. Yu Y. Cao D. Sun M. McLean J. R. Hollier B. G. (2012). Loss of breast epithelial marker hCLCA2 promotes epithelial-to-mesenchymal transition and indicates higher risk of metastasis. *Oncogene* 31 2237–2246. 10.1038/onc.2011.392 21909135 PMC4154589

[B57] Wang J. N. Yang Q. Yang C. Cai Y. T. Xing T. Gao L. (2020). Smad3 promotes AKI sensitivity in diabetic mice via interaction with p53 and induction of NOX4-dependent ROS production. *Redox Biol.* 32:101479. 10.1016/j.redox.2020.101479 32143149 PMC7058410

[B58] Wu T. Wang H. Xin X. Yang J. Hou Y. Fang M. (2020). An MRTF-A-Sp1-PDE5 axis mediates Angiotensin-II-Induced cardiomyocyte hypertrophy. *Front. Cell Dev. Biol.* 8:839. 10.3389/fcell.2020.00839 33015041 PMC7509415

[B59] Wu X. Dong W. Zhang T. Ren H. Wang J. Shang L. (2020). Epiregulin (EREG) and myocardin related transcription factor A (MRTF-A) form a feedforward loop to drive hepatic stellate cell activation. *Front. Cell Dev. Biol.* 8:591246. 10.3389/fcell.2020.591246 33520984 PMC7843934

[B60] Xu Y. Sengupta P. K. Seto E. Smith B. D. (2006). Regulatory factor for X-box family proteins differentially interact with histone deacetylases to repress collagen alpha2(I) gene (COL1A2) expression. *J. Biol. Chem.* 281 9260–9270. 16464847 10.1074/jbc.M511724200 PMC1434794

[B61] Yamaguchi T. Cubizolles F. Zhang Y. Reichert N. Kohler H. Seiser C. (2010). Histone deacetylases 1 and 2 act in concert to promote the G1-to-S progression. *Genes Dev.* 24 455–469. 10.1101/gad.552310 20194438 PMC2827841

[B62] Yang G. Weng X. Zhao Y. Zhang X. Hu Y. Dai X. (2017). The histone H3K9 methyltransferase SUV39H links SIRT1 repression to myocardial infarction. *Nat. Commun.* 8:14941. 10.1038/ncomms14941 28361889 PMC5381011

[B63] Yang X. J. Seto E. (2008). The Rpd3/Hda1 family of lysine deacetylases: from bacteria and yeast to mice and men. *Nat. Rev. Mol. Cell Biol.* 9 206–218. 10.1038/nrm2346 18292778 PMC2667380

[B64] Yang Y. Li Z. Guo J. Xu Y. (2020a). Deacetylation of MRTF-A by SIRT1 defies senescence induced down-regulation of collagen type I in fibroblast cells. *Biochim. Biophys. Acta Mol. Basis Dis.* 1866:165723. 10.1016/j.bbadis.2020.165723 32061777

[B65] Yang Y. Yang G. Yu L. Lin L. Liu L. Fang M. (2020b). An interplay between MRTF-A and the histone acetyltransferase TIP60 mediates hypoxia-reoxygenation induced iNOS transcription in macrophages. *Front. Cell Dev. Biol.* 8:484. 10.3389/fcell.2020.00484 32626711 PMC7315810

[B66] Yang Y. Wang H. Zhao H. Miao X. Guo Y. Zhuo L. (2021). A GSK3-SRF axis mediates Angiotensin II induced endothelin transcription in vascular endothelial cells. *Front. Cell Dev. Biol.* 9:698254. 10.3389/fcell.2021.698254 34381779 PMC8350349

[B67] Zeisberg E. M. Tarnavski O. Zeisberg M. Dorfman A. L. McMullen J. R. Gustafsson E. (2007). Endothelial-to-mesenchymal transition contributes to cardiac fibrosis. *Nat. Med.* 13 952–961. 10.1038/nm1613 17660828

[B68] Zhang Y. Wang H. Song M. Xu T. Chen X. Li T. (2020). Brahma-related gene 1 deficiency in endothelial cells ameliorates vascular inflammatory responses in mice. *Front. Cell Dev. Biol.* 8:578790. 10.3389/fcell.2020.578790 33330454 PMC7734107

[B69] Zhang Z. Chen B. Zhu Y. Zhang T. Zhang X. Yuan Y. (2021). The Jumonji domain-containing histone demethylase homolog 1D/lysine demethylase 7A (JHDM1D/KDM7A) is an epigenetic activator of RHOJ transcription in breast cancer cells. *Front. Cell Dev. Biol.* 9:664375. 10.3389/fcell.2021.664375 34249916 PMC8262595

[B70] Zhao H. Zhang Y. Xu X. Sun Q. Yang C. Wang H. (2021). Sall4 and myocd empower direct cardiac reprogramming from adult cardiac fibroblasts after injury. *Front. Cell Dev. Biol.* 9:608367. 10.3389/fcell.2021.608367 33718351 PMC7953844

[B71] Zhou B. von Gise A. Ma Q. Hu Y. W. Pu W. T. (2010). Genetic fate mapping demonstrates contribution of epicardium-derived cells to the annulus fibrosis of the mammalian heart. *Dev. Biol.* 338 251–261. 10.1016/j.ydbio.2009.12.007 20025864 PMC2815244

